# The Physicochemical Properties of TEMPO-Mediated Oxidized Xanthan Gum and Their Contribution to the Stability of Acidified Milk Drinks

**DOI:** 10.3390/foods15081363

**Published:** 2026-04-14

**Authors:** Jun Liu, Yonggang Zhang, Yanmin Zhang, Wei Wang, Yang Liu, Yusen Wu, Jiajie Luo, Siduo Zhou, Xueqian Dong

**Affiliations:** 1 Shandong Key Laboratory of Healthy Food Resources Exploration and Creation, School of Food Science and Engineering, Qilu University of Technology, Jinan 250353, China; 15092985145@163.com (J.L.); zyg8500@126.com (Y.Z.); zhangyanmin201314@163.com (Y.Z.); wangwei87@qlu.edu.cn (W.W.); liuyang0219@hotmail.com (Y.L.); boogiepop_w@163.com (Y.W.); jjieluo@163.com (J.L.); 2 Shandong Research and Design Institute of Food & Fermentation Industry, Qilu University of Technology, Jinan 250013, China

**Keywords:** xanthan gum, TEMPO oxidation, acidic milk beverage

## Abstract

Acidified milk drinks (AMDs) are susceptible to protein aggregation and phase separation during production and storage, and xanthan gum (XG) is limited in application due to poor compatibility with milk proteins under acidic conditions. This study sought to improve the stabilizing performance of XG in AMDs through TEMPO-mediated oxidation. A series of oxidized xanthan gum (OXG) with different oxidation degrees were prepared by the TEMPO/NaClO/NaBr system, whose physicochemical properties were characterized, and the stabilizing effect and mechanism in AMDs were investigated. The results showed that high-degree OXG significantly improved the stability of AMDs, with the centrifugal sedimentation rate reduced from 8.93% to 2.22% and the zeta potential decreased from −32.6 mV to −37.9 mV, achieving a stabilizing effect comparable to that of sodium carboxymethyl cellulose (CMC). OXG could form uniform protein–polysaccharide aggregates with milk proteins, which may help inhibit phase separation. This study confirms that TEMPO oxidation can directionally regulate the structure and physicochemical properties of XG, enhancing its stabilizing effect in AMDs, which provides a new technical approach and theoretical basis for polysaccharide modification and the stabilization of acidic protein drinks.

## 1. Introduction

AMDs refer to a type of milk beverage product made through direct acidification or fermentation, such as yogurt or blended yogurt. [1]. These products are highly favored by consumers for their unique flavor and health-promoting benefits, which has spurred their large-scale industrial production [2]. According to recent reports, the global market for acidic dairy beverages is projected to reach USD 10.6 billion by 2026 and USD 18.4 billion by 2033, with a compound annual growth rate (CAGR) of 8.2% during the forecast period (2026–2033) [3]. The pH value of AMDs is usually between 3.4 and 4.6. The proteins in cow’s milk mainly exist in the form of casein micelles, which account for about 80% of the total protein content. At neutral pH, the milk system remains stable due to the spatial repulsion provided by the extension conformation of κ-casein. However, upon acidification to approximately pH 4.6, the κ-casein conformation collapses, which weakens the spatial repulsion and triggers the aggregation and precipitation of casein micelles [4]. This aggregation and precipitation render the product susceptible to physical instability issues, including protein flocculation, whey separation, and sedimentation, which directly impact product quality and consumer acceptance. At present, there are three strategies to address the phase separation problem caused by the aggregation of casein in acidic environments. The first one is to add a single or several polysaccharide stabilizers, such as gelatin and carboxymethyl cellulose (CMC), in the AMD. This method has received high scientific attention and is also frequently used in industrial production [5]. The second is to modify the protein molecules themselves, for instance, through enzymatic treatment, to reduce their precipitation in acidic environments [6]. The third strategy involves optimizing processing and formulation parameters including protein content, homogenization pressure, and acidification rate, to indirectly enhance stability by modulating the system environment, thereby providing an optimized pathway for industrial production [7,8].

The quality of AMDs is influenced by a multitude of factors. A systematic understanding of these factors is crucial for developing improved AMD products with greater consumer appeal. The first is the characteristics of the raw milk, such as protein composition, initial casein micelle size, and the extent of heat treatment, all of which influence protein aggregation behavior. Appropriate heat treatment can induce the denaturation of whey proteins and their combination with casein to form a denser gel network, thereby improving product stability [9]. The second factor is processing parameters, including the previously mentioned homogenization pressure, control of acidification rate, etc. Generally, slow acidification promotes the formation of small protein aggregates, whereas rapid acidification tends to cause the precipitation of large particles. The third factor is formulation parameters, including not only the final pH value but also the milk solid content, stabilizer types, and other variables. An increase in milk solid content can improve rheological properties and significantly affect its microstructure, texture, and sensory characteristics [5]. The fourth factor is the sterilization method. Compared with pasteurization (~72 °C, 15 s), ultra-high temperature sterilization (135–150 °C, 1–10 s) can extend the shelf life to 9–12 months [10]. In conclusion, the stability of AMDs is a complex issue governed by multiple factors. A profound understanding of how these factors exert their influence is of great significance for optimizing product formulations and developing high-quality products.

The use of colloidal polysaccharides to stabilize AMDs has garnered considerable research attention. XG is widely used as a food thickener and stabilizer due to its excellent rheological properties [11]. XG is an anionic polysaccharide produced by the fermentation of Xanthomonas campestris. Its main-chain structure is similar to that of cellulose and has trisaccharide side chains. The side chains are wrapped around the main chain via hydrogen bonds, giving it a unique rigid rod-like double-helix conformation in aqueous solution [12,13]. This structure enables XG to increase the solution viscosity significantly at low concentrations and exhibits remarkable shear-thinning behavior, effectively suspending particles and preventing precipitation [14]. However, in milk beverage products, XG is usually not used alone and needs to be compounded with other polysaccharide food additives (such as pectin, sodium carboxymethyl cellulose, etc.) to achieve a stabilizing effect. This limitation stems from the unique rigid structure of XG, which prevents it from effectively wrapping around proteins through flexible chain entanglement in a neutral environment. Its high negative charge density and low hydrophobicity hinder the formation of a stable polysaccharide–protein system via hydrophobicity interactions or charge complementarity under these conditions [15]. This incompatibility can lead to thermodynamic incompatibility with milk proteins, potentially inducing depletion flocculation and, consequently, phase separation in the milk system [16]. During the acid adjustment process, as the pH value decreases, the negative charge on the surface of milk protein gradually reduces, making it prone to aggregation. Therefore, the depletion flocculation induced by XG and the charge-shielding effect on casein caused by acidification are superimposed, further exacerbating the separation and precipitation of milk proteins [17].

Chemical modification of XG is a key strategy to enhance its functional properties and broaden its application potential. By introducing or altering functional groups, it is possible to directionally enhance the hydrophilicity of XG, modify its charge distribution, or adjust its molecular conformation, thereby improving its physicochemical properties and processing performance. For instance, Bhardwaj et al. modified XG via amidation, which significantly enhanced its water-binding capacity and gel-network stability. The resulting drug-loaded gel beads exhibited excellent drug encapsulation and sustained release properties [18]. Zhang et al. acid-hydrolyzed XG and added XG derivatives to gluten-free dough to make bread. The results showed that XG derivatives had a lower degree of branching, and lower pyruvic acid and acetyl content, which could significantly increase the thickness of fermented dough, improve the specific volume of bread, and reduce the hardness of bread, thus improving the quality of gluten-free bread [19]. Although extensive research on the chemical modification of polysaccharides has been conducted at present, the existing modification techniques mostly focus on the optimization of a single function. In the food field, there are deficiencies in the research on the interaction mechanism between modified polysaccharides and food components. Furthermore, some modification methods rely on harsh chemical reagents, require stringent reaction conditions, or lack environmental friendliness, making it difficult to meet the industrial demands for “mild preparation and safety” of food additives.

The 2,2,6,6-tetramethylpiperidiny-1-oxide (TEMPO)-mediated oxidation method has been widely employed for modifying polysaccharides such as starch and cellulose, owing to its high selectivity and controllability [20,21]. TEMPO oxidation can oxidize the primary hydroxyl group at the C6 position of polysaccharides to a carboxyl group without affecting other functional groups, thereby tailoring their specific properties. Pei et al. [22] reported that TEMPO-oxidized oligochitosan achieved an oxidation degree of 80.77%. Compared to the untreated polysaccharide, the TEMPO-oxidized derivative exhibited a lower molecular weight and significantly enhanced hygroscopicity, moisture retention, and antioxidant capacity. Aaen et al. [23] demonstrated that TEMPO oxidation endows cellulose nanofibers (CNF) with negatively charged surfaces, and their rheological properties can be modulated through interactions with other food components, positioning them as promising fat replacers. These studies indicate that the application of TEMPO oxidation in polysaccharide modification is already quite mature. However, research gaps remain in the targeted oxidation of XG, particularly concerning the relationship between the oxidation degree and its stabilizing efficacy in acidic dairy beverages, and the potential for such modified XG to replace compounded commercial stabilizers.

The TEMPO oxidation method demonstrates broad application prospects in the field of polysaccharide modification due to its high selectivity and controllability. However, existing research mainly focuses on the modification of common polysaccharides such as starch and cellulose and their applications in materials science or drug delivery. Systematic studies on TEMPO oxidation of XG are still relatively scarce. Although XG is widely used as a food thickener due to its excellent rheological properties, its poor compatibility with milk proteins in acidic environments and the tendency to cause phase separation limit its use alone in acidic milk beverages. Currently, although there are studies on the chemical modification of xanthan gum, there is a lack of systematic data support and mechanism exploration in the research on TEMPO oxidation of XG to solve the stability problem of AMDs. The novelty of this study lies in the first systematic investigation of how the degree of TEMPO oxidation modulates the molecular structure and physicochemical properties of XG, and how these changes correlate with its stabilizing performance in acidic milk beverages. This aims to reveal the microscopic mechanism of OXG stabilizing milk proteins. Based on previous research and theoretical considerations, we hypothesized that increasing the carboxyl content of XG through TEMPO oxidation would: (1) enhance its electrostatic adsorption to casein under acidic conditions, and (2) modify its rigid structure by increasing molecular chain flexibility. These changes were expected to enable more uniform encapsulation of protein particles, thereby effectively inhibiting protein aggregation and phase separation. To verify these hypotheses, this study prepares different oxidation degrees of OXG through the TEMPO/NaClO/NaBr oxidation system and characterizes the functional groups, crystal structure, molecular weight, viscosity and other physicochemical properties of OXG and its derivatives using various technical means such as FT-IR, XRD, GPC and rheological analysis. Adding XG and its OXG derivatives to AMDs as stabilizers, the stability effect and mechanism of OXG on milk proteins in the AMD system are investigated through indicators such as centrifugal precipitation rate, turbidity, zeta potential, particle size distribution, rheological properties and microscopic structure (CLSM, SEM). This study aims to provide new strategies for resolving the stability issues of XG in AMDs and to establish a theoretical foundation for the modification of polysaccharides and their application in acidic protein beverage systems.

## 2. Materials and Methods

Commercial xanthan gum was procured from Shandong Fufeng Group (Linyi, China). 2,2,6,6-tetramethylpiperidiny-1-oxide, sodium bromide and Fast Green FCF were obtained from Shanghai Aladdin Biochemical Technology Co., LTD. (Shanghai, China). Sodium hypochlorite (available chlorine ≥ 6%) was purchased from Tianjin Comio Chemical Reagent Co., LTD. (Tianjin, China). Anhydrous ethanol, sodium hydroxide, hydrochloric acid, sulfuric acid, sodium tetraborate, carbazole, glucuronic acid, and sodium nitrate were all obtained from Sinopharm Group Chemical Reagent Co., LTD. (Shanghai, China). Anjia skimmed milk powder, food-grade sodium carboxymethyl cellulose, food-grade potassium sorbate and food-grade citric acid were all purchased from the local market in Jinan, China. Unless otherwise specified, all the above reagents are of analytical grade. The experimental water is deionized water, and the chromatographic experimental water is Milli-Q ultrapure water.

### 2.1. Preparation of TEMPO-Oxidized Xanthan Gum (OXG)

**Figure 1:**
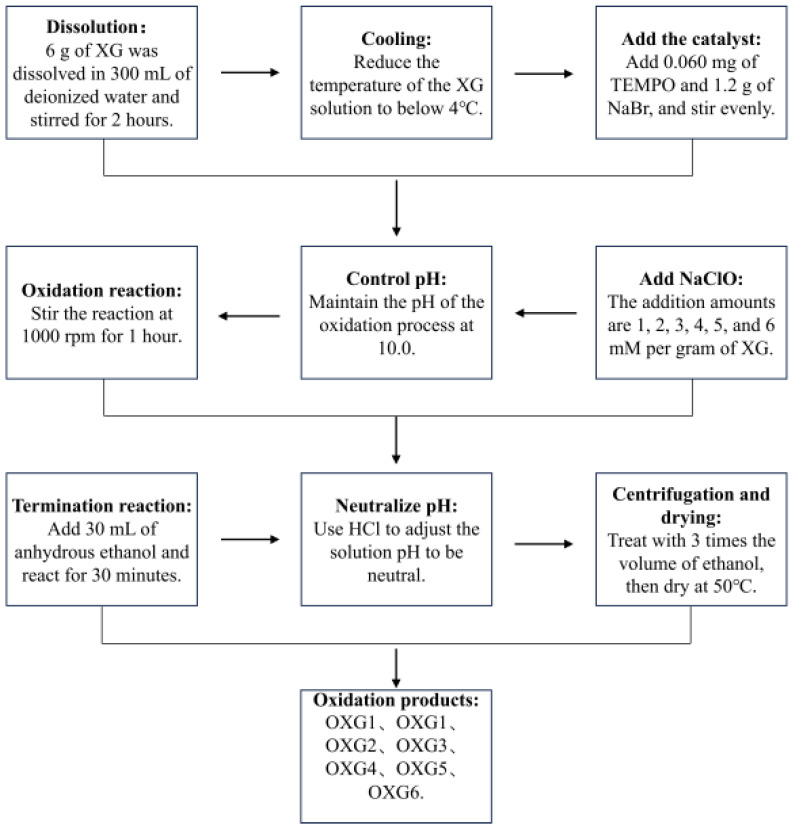
Flowchart of TEMPO oxidation of xanthan gum.

OXG was prepared following the method of Suh et al. [24] with slight modifications. XG (6 g) was accurately weighed and dissolved in 300 mL of deionized water at room temperature and stirred at 1000 rpm for 2 h using an OS20-S Electric Stirrer (DLAB, Beijing, China). The resulting solution was then cooled in an ice-water bath to maintain the temperature below 4 °C. Subsequently, TEMPO (60 mg) and NaBr (1.2 g) were accurately weighed, respectively, each dissolved in 5 mL of deionized water. These solutions were then added sequentially to the XG solution and mixed thoroughly. A predetermined volume of sodium hypochlorite solution (pH 10.0) was then added, and the mixture was stirred at 1000 rpm for 1 h using the electric stirrer. During the reaction, the pH value of the reaction system was adjusted and maintained at 10.0 using 1 mol/L NaOH. After the reaction, 30 mL of anhydrous ethanol was added, and the mixture was stirred for an additional 30 min to terminate the reaction. The pH of the solution was then adjusted to 7.0 using 1 mol/L HCl. The product was precipitated by adding three volumes of anhydrous ethanol, and the resulting precipitate was collected and washed repeatedly with ethanol. The final oxidized xanthan gum sample was obtained after drying at 50 °C. The process flow diagram for sample preparation is shown in Figure 1. The amount of sodium hypochlorite added is a key variable that regulates the physicochemical properties of the resulting OXG. Preliminary experiments showed that varying the NaClO addition from 1 to 6 mmol/g XG resulted in products with significantly different apparent viscosities. To systematically investigate the influence of NaClO dosage, six levels were selected: 1, 2, 3, 4, 5, and 6 mmol/g XG. The corresponding oxidized xanthan gum samples were sequentially named OXG1, OXG2, OXG3, OXG4, OXG5, and OXG6. OXG1–OXG6 were prepared in separate batches under identical reaction conditions. To ensure reproducibility, six parallel preparation replicates were conducted for each oxidation level.

### 2.2. Viscosity Measurements

The viscosity of the sample was determined by referring to the method described by Hu et al. [25] with slight modifications. XG and OXG were dissolved in deionized water to prepare 1.0% (*w*/*v*) solutions. The solutions were stirred at 1200 rpm for 1 h at room temperature to ensure complete dissolution and then allowed to stand for 10 min. Viscosity was measured at 25 °C and 60 rpm for 1 min using a Brookfield DV-2T rotational viscometer equipped with Rotor No. 3 (Brookfield, New York, NY, USA). For AMD samples, viscosity was determined after 1 day of storage at 25 °C and 100 rpm for 0.5 min using spindle No. 2. Each sample was measured in triplicate, and the mean ± standard deviation was calculated.

### 2.3. Determination of Carboxyl Group Substitution Degree (DS) and Uronic Acid Content

The DS was determined following the method of Shibakami et al. [26] with slight modifications. An accurately weighed polysaccharide sample (0.1 g) was completely dissolved in 50 mL of deionized water. The pH of the solution was adjusted to 3.2 using 0.1 mol/L HCl, and it was then titrated with 0.01 mol/L NaOH standard solution to an endpoint of pH 7.0. The volume of NaOH consumed was recorded. DS was calculated according to Formula (1) [26].

(1) $$A=C_{NaOH}\times V_{NaOH},DS=\frac{162.16\times A}{m-44\times A}$$

where *A* is the molar amount of sodium hydroxide consumed in the titration reaction, mol; *V_NaOH_* is the volume of NaOH solution consumed by titration, mL; *C_NaOH_* is the molar concentration of NaOH in the titration reaction, mol/L; *m* is the mass of the XG sample, g.

Uronic acid content was determined using the sulfuric acid–carbazole method [27]. A total of 1 mL of glucuronic acid standard solutions (10, 20, 30, 40, 50, 60, and 70 μg/mL, respectively) was pipetted into stoppered test tubes. Subsequently, 5 mL of sulfuric acid-borax reagent (4.77 g of sodium tetraborate dissolved in 500 mL of sulfuric acid) was added to each tube. The mixture was vortexed thoroughly and then heated in a boiling water bath for 10 min. After cooling to room temperature, 0.2 mL of carbazole reagent (0.125 g of carbazole dissolved in 100 mL of anhydrous ethanol) was added, and the mixture was heated again in boiling water for another 15 min. After cooling once more, the absorbance of the mixture was measured at 530 nm using a TU-1900 ultraviolet–visible (UV-Vis) spectrophotometer (PUXI general instrument, Beijing, China). A standard curve was plotted with the concentration of the glucuronic acid standard solution (μg/mL) on the *X*-axis and absorbance on the *Y*-axis. The 250 μg/mL sample solution was processed following the same procedure, and its absorbance was measured. The glucuronic acid content of the sample was calculated from the standard curve. Each OXG sample with different oxidation degrees was analyzed in triplicate for both assays.

### 2.4. Molecular Weight Determination

The molecular weight (M_w_) of the samples was determined by gel permeation chromatography (GPC) following the method of Xu et al. [28] with slight modifications. Samples were prepared as 1 mg/mL solutions in the mobile phase and filtered through a 0.22 μm membrane prior to analysis. Analysis was performed on an Agilent 1260 Infinity II GPC/SEC (Agilent Technologies, Santa Clara, CA, USA) equipped with a differential refractive index detector and a two-angle laser light-scattering detector. Separation was achieved on a PL aquagel-OH mix-h chromatographic column (300 × 7.5 mm, 8 μm, Agilent Technologies, USA) eluted with an aqueous NaNO_3_ solution (1 mg/mL) at a flow rate of 1 mL/min. The column temperature was maintained at 30 °C, and the injection volume was 0.1 mL. Each OXG sample with different oxidation degrees was determined in triplicate, and the average value was taken as the molecular weight result.

### 2.5. Fourier Transform Infrared (FT-IR) Spectroscopy

FT-IR spectra were recorded using the method of Zhao et al. [29] with slight modifications. Dried sample (1 mg) and KBr (150 mg) were accurately weighed, mixed, and ground thoroughly in an agate mortar. The homogeneous mixture was then pressed into a pellet for analysis. Spectra were recorded over a range of 4000–400 cm^−1^ using a Bruker TENSOR II Fourier Transform Infrared Spectrometer (Bruker, Billerica, MA, USA) with 32 scans and a resolution of 4 cm^−1^. Each OXG sample with different oxidation degrees was analyzed in triplicate, and a representative spectrum was used for subsequent structural analysis.

### 2.6. X-Ray Diffraction (XRD) Analysis

XRD patterns were obtained following the method of Geng et al. [22] with slight modifications. The sample powder was evenly distributed onto the sample holder, and the surface was leveled with a glass plate. Analysis was performed using an Ultima IV X-ray diffractometer (Rigaku Corporation, Tokyo, Japan) over a 2θ range of 5° to 60°, with a scanning speed of 5°/min, operating at 40 kV and 40 mA. Each OXG sample with different oxidation degrees was analyzed in triplicate, and representative diffraction patterns were selected.

### 2.7. Rheological Property

The rheological properties of 1% (*w*/*v*) XG and OXG solutions were determined using an MCR-302 rheometer (Anton Paar, Graz, Austria) equipped with a 40 mm parallel plate, following the method of Geng et al. [30] with slight modifications. Amplitude sweep tests were conducted over a strain range of 0.01–10% to determine the linear viscoelastic region (LVR) for subsequent frequency sweep measurements. Frequency sweep tests were performed from 0.1 to 100 rad/s at a constant strain of 0.1% (within the LVR) to measure the storage modulus (G′) and loss modulus r (G″). The apparent viscosity was measured as a function of shear rate over a range of 0.1–100 s^−1^. All measurements were performed with a gap of 1 mm, and the temperature was maintained at 25 °C. For AMD samples, the apparent viscosity was measured as a function of shear rate from 0.1 to 1000 s^−1^ at 25 °C. Each sample was analyzed in triplicate, and the average values were used to plot rheological curves.

### 2.8. Zeta Potential and Particle Size Analysis

The zeta potential and particle size of the samples were analyzed following the method of Burken et al. [31] with slight modifications. The sample was diluted to 0.1 mg/mL and analyzed for zeta potential and nanoparticle size using a Zetasizer Nano ZS90 zeta potential and nanoparticle size analyzer (Malvern Instruments, Worcestershire, UK). For polysaccharide samples, the material was specified as polystyrene latex, with water as the dispersant and a refractive index (RI) of 1.59. For AMD samples, the material was defined as protein, with water as the dispersant and an RI of 1.45. The particle size distribution of AMD samples was determined using a Malvern 3000 laser particle size analyzer (Malvern Instruments, Worcestershire, UK). The particle size of AMDs was tested after being stored for 1 d. The solute shape is selected irregularly, the material is protein, and the refractive index is 1.529 [32]. All samples were analyzed in triplicate.

### 2.9. Preparation of AMDs

The AMD was prepared following the method of Zhao et al. [29] with slight modifications, according to the formulation detailed in Table 1. Skimmed milk powder (8.5 g) and the polysaccharide (1 g) were each dissolved in 50 mL of deionized water and hydrated overnight at 4 °C. The two solutions were then combined at 25 °C and stirred for 30 min. Then, 8 mL of 10% citric acid solution was slowly added for acid adjustment, and the final pH values of all AMD samples after acidification remained at 4.1 ± 0.1, which was within the industrial pH range of AMDs, and the pH values of AMD samples with different polysaccharides remained consistent after acidification treatment. Subsequently, potassium sorbate (0.6 g) was added, and the volume was made up to 200 mL with deionized water. The mixture was then homogenized using an AH-BASIC ultra-high-pressure homogenizer (Antos Nanotechnology Co., Ltd., Jiangsu, China) at 200 bar and 25 °C for 3 min. The resulting AMD samples were designated as CMC-AM, XG-AM, OXG1-AM, OXG2-AM, OXG3-AM, OXG4-AM, OXG5-AM, and OXG6-AM, based on the polysaccharide stabilizers used. For storage stability assessment, aliquots of AMDs were transferred into glass tubes and stored in a constant temperature chamber at 37 °C. Visual changes were recorded photographically at 1, 7, and 15 d of storage. Another set of AMDs was stored at 4 °C for subsequent analyses. CMC, a widely used stabilizer in acidic beverages, served as a positive control. The polysaccharide concentration in all AMD samples was fixed at 0.5% (*w*/*v*), which is within the typical industrial application range (0.2–0.6% *w*/*v*) for CMC to ensure both effective stability and desirable drinkability. AMD samples were prepared in triplicate for each polysaccharide stabilizer with different oxidation degrees, and all were used for subsequent stability tests.

### 2.10. Determination of Centrifugal Sedimentation Rate (CSR) and Turbidity

The CSR and turbidity of AMDs were measured following the method of Zhao et al. [21] with slight modifications. AMD samples (10 g) were centrifuged at 4000 rpm for 20 min at 25 °C. The supernatant was collected and diluted 20-fold with deionized water. The turbidity of the diluted supernatant was then measured at 550 nm using a TU-1900 ultraviolet–visible (UV-Vis) spectrophotometer (PUXI general instrument, Beijing, China). The centrifuge tube was then inverted for 5 min to drain the residual supernatant, and the mass of the remaining precipitate was weighed. The calculation formula for the centrifugal sedimentation rate is as follows:

(2) $$CSR\left(\%\right)=\frac{M_{1}}{M_{0}}\times 100\%$$

where *CSR* (%) represents the centrifugal sedimentation rate; *M*_0_ represents the weighed AMD mass, g; *M*_1_ represents the mass of the precipitate after centrifugation, g.

All samples were analyzed in triplicate, and the mean ± standard deviation was calculated.

### 2.11. Confocal Laser Scanning Microscopy (CLSM)

The microstructure of AMDs was observed using CLSM following the method of Aljewicz et al. [33] with slight modifications. Imaging was performed using a Leica SP8 confocal microscope (Leica Microsystems Inc., Heidelberg, Germany). In total, 1 mL of the sample was mixed thoroughly with 0.2 mL of Fast Green FCF solution (0.1 mg/mL aqueous solution), and an appropriate amount of the stained sample was transferred onto a microscope slide. A coverslip was placed over the sample, and observations were made with an excitation wavelength of 653 nm. Images were acquired at a resolution of 2048 × 2048 pixels. Five random regions of each sample were photographed, and representative images were selected for analysis.

### 2.12. Scanning Electron Microscopy (SEM)

The microstructure of AMDs was further examined using SEM, according to the method of Zhao et al. [29] with minor modifications. Freshly prepared AMD samples were rapidly frozen in liquid nitrogen for 1 h and then freeze-dried under vacuum for 48 h. The freeze-dried samples were mounted onto aluminum stubs using double-sided conductive adhesive tape and sputter-coated with gold. Imaging was performed using a JSM-6610LV benchtop scanning electron microscope (JEOL, Tokyo, Japan) at an accelerating voltage of 20 kV, a working distance of 8 mm, and a magnification of 500×. Five random regions of each sample were photographed, and representative images were selected for analysis.

### 2.13. Statistical Analysis

All samples were analyzed in triplicate for their physicochemical properties. The results were expressed as mean ± standard deviation. Statistical analysis was conducted using SPSS 23.0 (SPSS Inc., Chicago, IL, USA). Analysis of variance (ANOVA) and a significance test (*p* < 0.05) were employed to determine significant differences among the groups. Heatmap analysis was conducted in Origin 2021 (OriginLab Corporation, Northampton, MA, USA) using the Pearson correlation coefficient.

## 3. Results and Discussion

### 3.1. Structural Characterization of XG and OXG

The TEMPO oxidation method is an oxidative modification approach that selectively oxidizes the hydroxyl groups at the C-6 position of polysaccharides to carboxyl groups. Therefore, the degree of carboxyl substitution (DS) was used as an indicator to assess the extent of modification. In this study, the DS values of XG and OXG1-6 were directly determined by acid–base titration. As the carboxyl content of OXG after oxidation was considerably higher than that of native XG, the calculated DS values directly reflected the differences in carboxyl content among OXG samples with varying oxidation degrees. This facilitated the analysis of how the oxidation modification regulated the carboxyl substitution of XG. The results are shown in Table 2. The DS value of XG was 0.174 ± 0.004. With the increase in the amount of the oxidant NaClO, the DS increased substantially. The DS values for OXG1 to OXG6 were 0.185 ± 0.003, 0.192 ± 0.001, 0.211 ± 0.003, 0.233 ± 0.001, 0.248 ± 0.003, and 0.269 ± 0.001, respectively, suggesting that the proportion of hydroxyl groups in the XG structure oxidized to carboxyl groups increased with the degree of oxidation. An increase in the dosage of NaClO contributes to the elevation of the carboxyl substitution degree of oxidized polysaccharides, the core mechanism of which lies in the role of NaClO as a primary oxidant in the TEMPO-catalytic cycle. NaClO enables the in situ regeneration of the nitrosonium ion, the active species of TEMPO. A sufficient amount of NaClO ensures a sustained high concentration of the nitrosonium ion, which efficiently oxidizes more primary hydroxyl groups (at the C-6 position) to aldehyde intermediates and subsequently facilitates their conversion to carboxylate groups under the aqueous reaction conditions [34]. In Huang et al.’s research on the TEMPO oxidation modification of chitin, it was found that as the amount of NaClO added for oxidation increased from 8 mmol/g to 20 mmol/g, the carboxylic acid content of chitin rose from 1.33 mmol/g to 2.08 mmol/g. [35]. This is similar to the change pattern observed in this study where the carboxyl substitution degree of xanthan gum significantly increased with the increase in sodium hypochlorite addition. In addition, the yields of the oxidation reactions were determined for each of OXG1-6 through three independent batches of preparation. The yields of OXG1-6 were 96.2 ± 0.3%, 95.7 ± 0.2%, 95.5 ± 0.4%, 95.1 ± 0.2%, 94.4% ± 0.2%, and 94.0 ± 0.1%, respectively. The slight decrease in yield with increasing oxidation degree may be attributed to the loss of low-molecular-weight polysaccharide fragments, which remained soluble in the ethanol–water mixture during precipitation. The results indicate that the method allowed for the regulation of the oxidation degree while maintaining a good product recovery rate.

The results of the uronic acid content of the samples are also presented in Table 2. The standard curve of uronic acid is y = 0.0174x − 0.0536, R^2^ = 0.996. Based on this standard curve, the uronic acid content of native XG was determined to be 49.805 ± 0.065 μg/mL. Following TEMPO oxidation modification, the uronic acid content increased with the degree of oxidation. The uronic acid contents of OXG1-6 were 51.375 ± 0.025 μg/mL, 52.851 ± 0.021 μg/mL, 53.061 ± 0.043 μg/mL, 53.425 ± 0.013 μg/mL, 54.174 ± 0.021 μg/mL, and 54.747 ± 0.051 μg/mL, respectively. This trend is highly consistent with that observed for the DS values. This result indicates that the terminal hydroxyl groups in the XG structure were oxidized to carboxyl groups, which is the direct cause of the increase in uronic acid content. This further supports the directional modification effect of TEMPO oxidation on the functional groups of XG [36,37]. In the study by Delattre et al. [38], the uronic acid content of xanthan gum increased from 20% to 98% after TEMPO oxidation, which is consistent with the results of this experiment.

The molecular weight determination for the samples is shown in Table 2. The molecular weight of XG is 9.610 ± 0.080 × 10^6^ g/mol. The molecular weights of OXG1-6 were 7.660 ± 0.060 × 10^6^ g/mol, 5.830 ± 0.010 × 10^6^ g/mol, 2.980 ± 0.010 × 10^6^ g/mol, 1.420 ± 0.010 × 10^6^ g/mol, 1.280 ± 0.010 × 10^6^ g/mol, and 0.580 ± 0.010 × 10^6^ g/mol, respectively. The results showed that the molecular weight of OXG decreased progressively with the degree of modification. This decrease can be attributed to the cleavage of glycosidic bonds in the polysaccharide backbone and side chains, induced by the combined action of the oxidant NaClO and the alkaline reaction conditions. The oxidation degree and molecular weight show a negative correlation. Tamura et al. [39] found that after TEMPO oxidation modification of commercial starch and konjac gum, the molecular weight and degree of polymerization of polysaccharides decreased.

**Figure 2:**
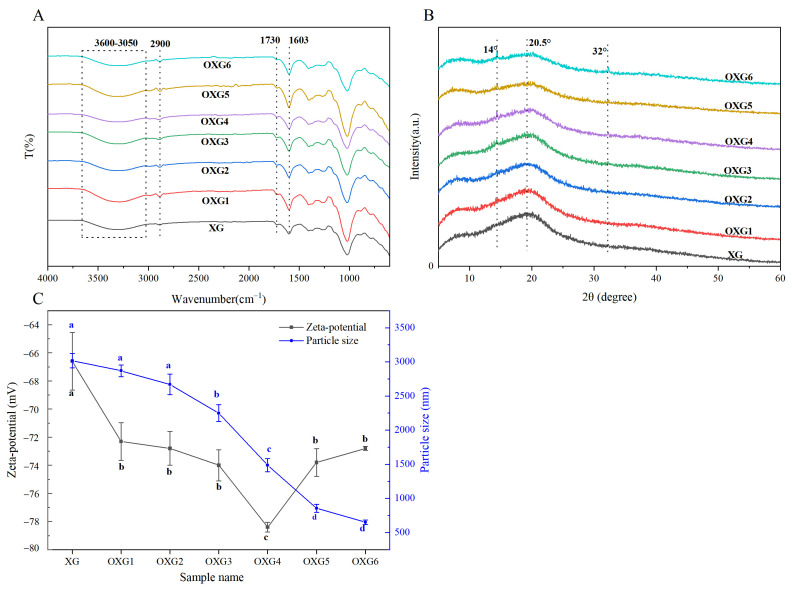
Xanthan gum and xanthan gum TEMPO oxidation FT-IR (**A**), XRD (**B**), zeta potential, and particle size distribution (**C**). XG represents xanthan gum, while OXG1, OXG2, OXG3, OXG4, OXG5, and OXG6, respectively, indicate xanthan gum with different degrees of TEMPO oxidation. Different lowercase letters indicate statistically significant differences within the group (*p* < 0.05).

Figure 2A shows the FT-IR spectra of XG and OXG. The strong, broad absorption band in the range of 3600–3050 cm^−1^ is attributed to the stretching vibration of -OH groups, while the band at 2920 cm^−1^ corresponds to the asymmetric stretching vibration of -CH_2_ [40]. The signals at 1603 cm^−1^ and 1730 cm^−1^ are associated with the C=O stretching of alkyl esters and the asymmetric stretching of carboxylate ions (-COO^-^), respectively. Both XG and OXG have carboxyl characteristic peaks. The results indicated that XG and OXG had similar FT-IR spectra.

X-ray diffraction is a powerful tool for characterizing the crystalline properties of polysaccharides. The XRD patterns of XG and OXG1-6 are presented Figure 2B. All polysaccharides exhibited a relatively broad diffraction peak centered at 20.5°, suggesting that XG and OXG have amorphous properties. With increasing oxidation degree, the diffraction intensity in the amorphous region decreased. This decrease in intensity may be explained by the fact that native XG contains numerous hydroxyl groups capable of forming dense hydrogen bonds, which link adjacent molecular chains or segments into relatively ordered, compact arrangements, giving rise to locally ordered regions [41]. Upon oxidation, the -OH groups at the C-6 position are converted to -COOH groups, which reduces the polysaccharide’s capacity to form hydrogen bonds. Furthermore, the electrostatic repulsive between the newly introduced carboxyl groups may disrupt the original local ordered structure, contributing to the further reduction in the intensity of amorphous diffraction peaks [42]. As DS increased, small, sharp crystalline peaks appeared at 14° and 32°. The emergence of these new peaks could be attributed to oxidative modification altering the polysaccharide chain conformation and changing the stacking mode [43]. In the study by Rakshit et al. [44] on XG modification, increasing the carboxyl content of the modified XG enhanced the polymer’s crystallinity, consistent with the results of this experiment.

### 3.2. Property Characterization of XG and OXG

The viscosity of the polysaccharide solution is presented in Table 2. The results indicated that the higher the sample’s DS, the lower its viscosity. In aqueous solution, the side chains of XG wind around the main chain in a reverse direction, stabilized by hydrogen bonds, to form a rigid, rod-shaped double helix. These molecules further associate through hydrogen bonds to form a polymer network that effectively immobilizes water, increasing internal friction and, consequently, imparting high viscosity to the solution [45]. TEMPO-mediated oxidation to some extent disrupts the structure of polysaccharide polymers, causing the glycosidic bonds of XG to break and the degree of polymerization to decrease, resulting in a reduction in viscosity [46]. Furthermore, the increase in the number of carboxyl groups at higher DS enhances electrostatic repulsion between XG chains. This repulsion disrupts intermolecular entanglements and weakens hydrogen bonding, both of which contribute to the observed viscosity reduction. Chen et al. [47] found in their study on the TEMPO-mediated oxidation of konjac glucomannan that the viscosity of the polysaccharide solution decreased significantly with increasing oxidation degree, attributed to the increase in the polymer’s carboxyl content.

**Figure 3:**
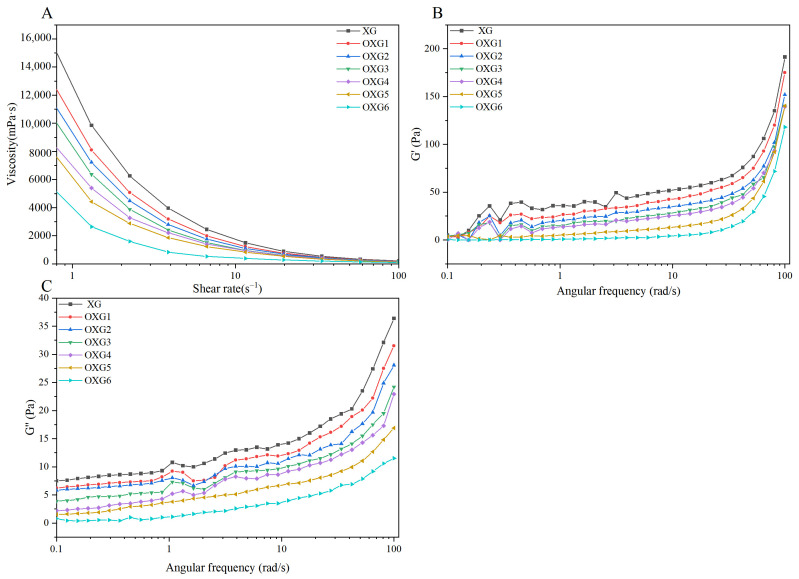
Rheological property determination of xanthan gum and TEMPO-oxidized xanthan gum. Apparent viscosity variation curves at different shear rates (**A**), energy storage modulus variation curves at different angular frequencies (**B**), and loss modulus variation curves (**C**). XG represents xanthan gum, while OXG1, OXG2, OXG3, OXG4, OXG5, and OXG6, respectively, indicate xanthan gum with different degrees of TEMPO oxidation.

The apparent viscosity of the polysaccharide solutions as a function of shear rate is shown in Figure 3A. For all samples, the apparent viscosity decreased continuously with increasing shear rate, a characteristic shear-thinning (pseudoplastic) behavior. This phenomenon could be attributed to the shear-induced disruption of intermolecular interactions and chain entanglements, which may align in the direction of flow, thereby potentially reducing resistance. Consistent with the viscosity data in Table 2, the apparent viscosity of OXG samples decreased progressively with increasing DS across the entire shear rate range. This is likely due to the conversion of primary hydroxyl groups to carboxyl groups, which reduces hydrogen bonding within and between XG molecules. Simultaneously, the increase in carboxyl groups enhances the electrostatic repulsion between molecules, which hinders the formation of double-helix aggregation and network structure through hydrogen bonds of XG molecules. Consequently, the molecular chains adopt a more extended and independent conformation, reducing flow resistance and macroscopically resulting in lower viscosity [48,49]. In addition, it was associated with the cleavage of glycosidic bonds during the oxidation of XG.

The storage modulus (G′) reflects the elastic behavior of the solution, while the loss modulus (G″) represents its viscous behavior. A decrease in G′ indicates a weakening of the elastic network, while a decrease in G″ signifies reduced viscosity and lower flow resistance. The G′ and G″ variation curves of the sample at different angular frequencies are shown in Figure 3B and Figure 3C, respectively. The G′ and G″ of XG and OXG increased with the increase in angular frequency, showing angular frequency dependence. Moreover, as DS increased, the G′ and G″ of OXG1-6 showed a decreasing trend, which was similar to the variation law of the apparent viscosity of the sample. This indicates that the elastic behavior of the solution system weakens, the viscosity decreases, and the flow resistance reduces. These observations further support the conclusion that TEMPO oxidation reduces chain entanglements, while the introduction of carboxyl groups enhances the overall fluidity of the system.

Zeta potential is a critical parameter governing the stability of colloidal systems. Research has found that when the zeta potential is above 30 mV or below −30 mV, electrostatic repulsion between particles reduces the likelihood of molecular aggregation [50]. As shown in Figure 2C, the zeta potential values of XG and OXG are negative, indicating that XG and OXG were anionic polysaccharides. The zeta potential of OXG was all lower than that of unmodified XG, showing an overall trend of first decreasing and then increasing. The zeta potential values of the OXG1-4 sample decreased with the increase in DS, which were −72.300 ± 1.300 mV, −72.800 ± 2.100 mV, −74.000 ± 2.400 mV, and −78.400 ± 0.400 mV, respectively. This initial decrease can be attributed to the introduction of a large number of -COO^−^ groups through TEMPO oxidation, which increases the net negative charge density on the OXG molecule, with OXG4 exhibiting the lowest zeta potential. The loosening of the molecular structure may also contribute to the exposure of additional charged groups [51]. With further oxidation, the zeta potentials of OXG5 and OXG6 were −73.800 ± 1.000 mV and −72.800 ± 0.100 mV, respectively. This slight increase might be related to the progressive destruction of the rigid double-helix structure, leading to more flexible or randomly coiled conformations. Concurrently, the polysaccharide chains undergo degradation, breaking into shorter segments. These conformational changes and chain shortening could alter the spatial distribution of the negatively charged carboxyl groups, potentially leading to the partial shielding of some charges. Although there are significant differences in carboxyl content, the measured zeta potential is not solely determined by the total number of carboxyl groups. It is also influenced by the degree of dissociation of surface carboxyl groups, the charge density, and, importantly, the conformational behavior of the polysaccharide chains in solution [52]. A similar trend in zeta potential with varying oxidation degree was observed by Ma et al. [53] for TEMPO-oxidized yeast β-glucans. They reported that the zeta potential initially decreased from −0.560 mV to −55.100 mV (OYG1 to OYG4) and then slightly increased to −51.200 mV at the highest oxidation degree (OYG5). The trend of zeta potential change was similar to that of OXG in this experiment, suggesting that the recovery of zeta potential at a high oxidation degree might be a complex process involving multiple factors.

The particle size distribution of the samples is shown in Figure 2C and Table 2. The mean particle size value of XG was 3016.000 ± 443.000 nm. The particle size values of OXG1-6 were 2870.000 ± 86.000 nm, 2669.000 ± 231.000 nm, 2249.000 ± 213.000 nm, 1488.000 ± 96.000 nm, 856.000 ± 58.000 nm, and 650.000 ± 32.000 nm, respectively. The mean particle size of OXG decreased progressively with increasing oxidation degree. For lower oxidation degrees (OXG1–OXG3), the reduction in particle size may be attributed to the introduction of carboxyl groups, which increases negative charge density and electrostatic repulsion. This repulsion disrupts interchain hydrogen bonds and hydrophobic interactions, leading to the disassembly of aggregated structures. With further oxidation (OXG4–OXG6), particle size decreased more substantially. The polydispersity index (PDI) of XG and OXG1-6 were 1.000, 1.000, 1.000, 0.869, 0.659, 0.586, and 0.478, respectively, indicating a significant improvement in the uniformity of the particle size distribution in the system. This substantial reduction in particle size at higher oxidation degrees likely results from the cleavage of main-chain glycosidic bonds, leading to the formation of lower molecular weight polysaccharides. Kaya [54] used TEMPO to oxidize cellulose to prepare cellulose nanofibers (CNF), and measured the particle size of CFM before and after oxidation. The results showed that the particle diameters of the fibers modified by TEMPO oxidation were much smaller than those of fibers obtained through chemical or mechanical processing alone. This result was consistent with the trend of the OXG particle size changes in this experiment.

### 3.3. Correlation Analysis

**Figure 4:**
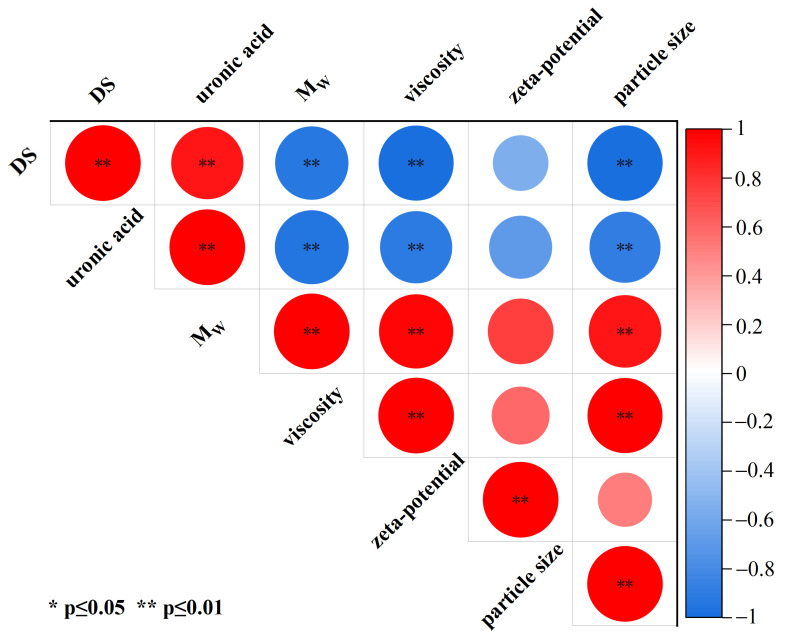
Pearson correlation coefficients among DS, uronic acid content, molecular weight, viscosity, zeta potential, and particle size of xanthan gum and TEMPO-oxidized xanthan gum. Red indicates a positive correlation, blue indicates a negative correlation, and the lighter the chroma, the smaller the correlation. “∗” and “∗∗” indicate statistical significance at the *p* ≤ 0.05 and *p* ≤ 0.01 levels, respectively.

A Pearson correlation analysis was conducted to evaluate the relationships among the six key parameters: DS, uronic acid content, molecular weight, viscosity, zeta potential, and particle size. The results are presented as a heatmap in Figure 4. In the heatmap, red and blue indicate positive and negative correlations, respectively, with color intensity reflecting the strength of the correlation. Specifically, DS was significantly positively correlated with glucuronic acid content (*p* ≤ 0.01), which not only confirms that TEMPO oxidation successfully converts the C6 hydroxyl groups of XG into carboxyl groups, but also suggests that the carboxyl content can be precisely controlled by controlling the amount of oxidant NaClO. DS was significantly negatively correlated with molecular weight, viscosity, and particle size (*p* ≤ 0.01). As discussed in Section 3.2, this can be explained by the non-specific attack of NaClO on glycosidic bonds, which leads to a reduction in molecular weight. This reduction in molecular weight, in turn, decreases the hydrodynamic volume and disrupts the chain entanglement network, ultimately leading to lower viscosity and smaller particle size. Uronic acid content was significantly negatively correlated with molecular weight, viscosity, and particle size (*p* ≤ 0.01), as this part has the same explanation as the significant negative correlation between DS and molecular weight, viscosity, and particle size mentioned above. Molecular weight was significantly positively correlated with viscosity and particle size (*p* ≤ 0.01), further underscoring that molecular size is the fundamental factor determining of the solution behavior of XG and its derivatives. A decrease in molecular weight reduces the hydrodynamic volume and weakens intermolecular entanglement, which macroscopically manifests as lower viscosity and smaller particle size. This correlation is further supported by the rheological curve of Figure 3. At high oxidation degrees (OXG5, OXG6), throughout the shear rate range, the apparent viscosity and modulus of high-oxidation-degree XG show lower values. Viscosity was also significantly positively correlated with particle size (*p* ≤ 0.01), confirming that in these polysaccharide systems, a larger hydrodynamic size is associated with higher solution viscosity. At the molecular level, particle size is a direct reflection of the hydrodynamic volume and the extent of intermolecular chain entanglement. Larger particle size means longer molecular chains and a more dense entanglement network, which can effectively bind water molecules and increase the flow resistance, resulting in high viscosity. The increase in TEMPO oxidation degree leads to glycosidic bond cleavage and enhanced electrostatic repulsion from newly introduced carboxyl groups. These changes disrupt interchain entanglements, causing a significant reduction in particle size and weakening of the network structure, which in turn decreases viscosity. Zeta potential has no significant correlation with DS, glucuronic acid content, molecular weight, viscosity, and particle size (*p* > 0.05). This lack of correlation suggests that the zeta potential is not governed solely by the total carboxyl content. Instead, it is a complex parameter influenced by a combination of factors, including molecular conformation, chain flexibility, and the spatial distribution of charges. At high oxidation degrees (OXG5, OXG6), severe chain degradation and conformational changes may alter the spatial distribution of the charged groups, potentially leading to the partial shielding of some originally exposed charges.

### 3.4. Centrifugal Sedimentation Rate (CSR) and Turbidity of AMDs

CSR is a key indicator for assessing the physical stability of AMDs. The lower its value, the better the stability of milk protein in an acidic environment. It is worth noting that CSR is also often used as a rapid evaluation index for assessing the stability of natural sedimentation in AMD systems [55]. The results are shown in Table 3. The CSR of AMDs stabilized with XG was 8.930 ± 0.070%. The CSR of the samples with OXG1-6 added as the stabilizer was 6.850 ± 0.060%, 5.990 ± 0.140%, 5.480 ± 0.080%, 4.660 ± 0.040%, 2.540 ± 0.020%, and 2.220 ± 0.010% respectively, while the CSR with CMC added as the stabilizer was 2.050 ± 0.260%. These results indicate that AMDs containing native XG exhibited the highest CSR, indicating the poorest stability against sedimentation. In contrast, when OXG5 or OXG6, which have higher DS, were used, the CSR values were significantly lower, approaching that of the CMC-stabilized sample, and indicating a substantial improvement in stability. The zeta potential values of AMDs were also measured, and the results are shown in Table 3. The zeta potential values of XG and OXG1-6 were −32.600 ± 0.300 mV, −33.500 ± 0.100 mV, −34.300 ± 0.200 mV, −34.900 ± 0.200 mV, −35.900 ± 0.100 mV, −36.700 ± 0.100 mV, and −37.900 ± 0.300 mV, respectively. The CMC-AM sample exhibited a zeta potential of −38.000 ± 0.500 mV. The improved stability and more negative zeta potential of AMDs with high-DS OXG may be partly explained by the increased negative charge on the polysaccharide. Under acidic conditions, these negatively charged OXG molecules may adsorb onto the surface of protein particles via electrostatic interactions. Such adsorption could contribute to the formation of a charged protective layer [56], which might enhance electrostatic repulsion and steric hindrance between protein particles, thereby preventing aggregation and reducing CSR. OXG6-AM showed CSR and zeta potential values comparable to those of CMC-AM, with no significant difference. In contrast, the CSR values for OXG1-AM to OXG5-AM were significantly higher, and their zeta potentials significantly less negative than those of CMC-AM. These results suggest that OXG with a sufficiently high oxidation degree achieves a level of inhibition against protein precipitation comparable to that of CMC. It has been previously reported that CMC with a higher degree of carboxymethyl substitution provides better stabilization in AMDs than CMC with a lower substitution degree. This is because CMC with a higher DS will generate a stronger electrostatic repulsion between CMC and casein complexes [57], which is similar to the results of this experiment.

The turbidity method measures light-scattering properties to estimate the concentration of dispersed particles in the solution. A high turbidity value indicates good solution stability, while a low value suggests greater protein flocculation or deposition in the system [58]. The turbidity results for the AMD samples are shown in Table 3. The turbidities of AMDs with XG and OXG1-6 added as stabilizers were 0.040 ± 0.020, 0.700 ± 0.040, 0.910 ± 0.030, 0.950 ± 0.030, 1.060 ± 0.010, 1.110 ± 0.020, and 1.330 ± 0.020, respectively, showing an overall gradual increase. The CMC-AM sample had a turbidity of 1.110 ± 0.060. This trend is consistent with the CSR results. In AMDs, the greater the DS of added OXG, the higher the turbidity value. This can be explained by the fact that high-DS OXG, carrying more negative charges, may form strong electrostatic interactions with positively charged casein, favoring the formation of smaller, more uniformly dispersed protein–polysaccharide aggregates. Such aggregates effectively inhibit protein flocculation and sedimentation. Furthermore, their small size and uniform distribution enhance light scattering, resulting in a higher turbidity value. These results indicate that milk proteins maintain good suspension stability in AMD systems containing high-DS OXG, with macroscopic phase separation being significantly suppressed. The turbidity of OXG6-AM was significantly higher than that of CMC-AM, suggesting that the protein–polysaccharide complexes formed with OXG6 were finer and more uniformly dispersed, leading to stronger light-scattering ability. The turbidity values for OXG4-AM and OXG5-AM were not significantly different from that of CMC-AM, whereas those for OXG1-AM to OXG3-AM were significantly lower. This suggests that as the oxidation degree increases, the ability of OXG to form stable complexes gradually strengthens and shows a tendency to surpass CMC.

### 3.5. The Influence of Different Stabilizers on the Appearance Stability of AMDs During Storage

**Figure 5:**
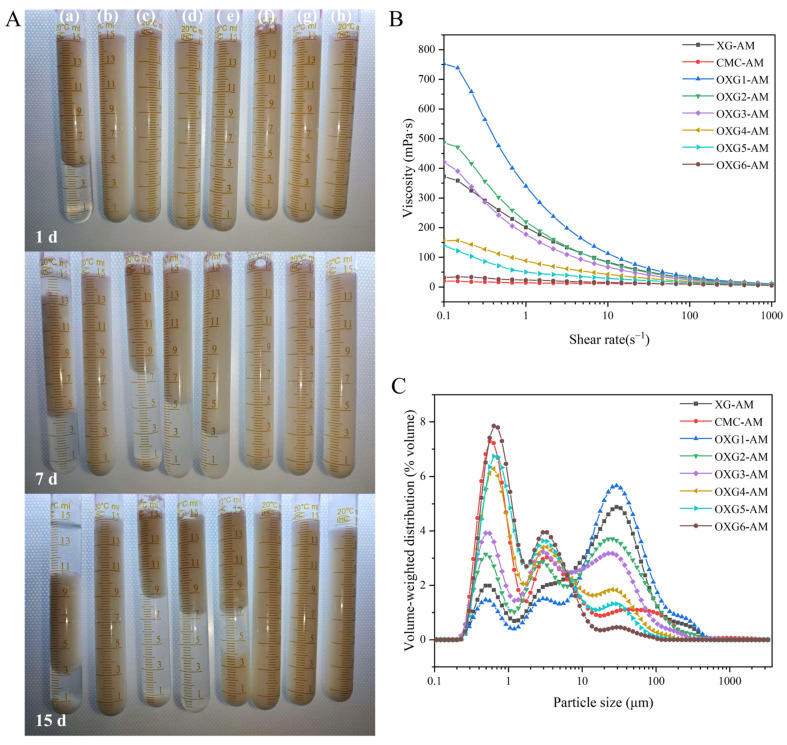
Appearance states of AMDs with different stabilizers added after storage for 1 d, 7 d, and 15 d (**A**), rheological curves of AMDs with different polysaccharides added (**B**), and particle size distribution of AMDs with different polysaccharides added (**C**). In (**A**,**B**), XG-AM represents AMDs with xanthan gum added, CMC-AM represents AMDs with sodium carboxymethyl cellulose added, and OXG1-AM, OXG2-AM, OXG3-AM, OXG4-AM, OXG5-AM, and OXG6-AM represent AMDs with xanthan gum of different oxidation degrees of TEMPO added. From left to right in (**C**), they are XG-AM (a), CMC-AM (b), OXG1-AM (c), OXG2-AM (d), OXG3-AM (e), OXG4-AM (f), OXG5-AM (g), and OXG6-AM (h) respectively; from top to bottom are the appearance states of AMDs with storage times of 1 d, 7 d, and 15 d respectively.

To evaluate the storage stability, the visual appearance of AMD samples was recorded after 1, 7, and 15 d of storage at 37 °C, as shown in Figure 5A. Storage at 37 °C was conducted as an accelerated stability test to simulate potential high-temperature conditions encountered during commercial distribution and to enable a rapid assessment of stabilizer efficacy. When stored for 1 d, XG-AM already showed signs of whey separation, while all other samples appeared as a uniform emulsion without visible stratification or sedimentation. When stored for 7 d, whey separation was more pronounced in XG-AM. OXG1-AM, OXG2-AM, and OXG3-AM began to exhibit slight sedimentation but retained a degree of homogeneity. All other AMD samples maintained good uniformity. When stored for 15 d, XG-AM exhibited severe stratification and sedimentation. The samples stabilized with OXG1, OXG2, and OXG3 also showed varying degrees of whey separation and sedimentation. In contrast, OXG4-AM, OXG5-AM, OXG6-AM and CMC-AM remained homogenous with no visible signs of instability. In summary, the long-term storage stability of AMD samples stabilized with CMC and with OXG (especially OXG4, OXG5, OXG6) was markedly superior to that of XG-AM. Samples with lower oxidation degrees (OXG1-AM, OXG2-AM, OXG3-AM) could not maintain stability over extended storage and were prone to severe phase separation and sedimentation. Conversely, OXG4-AM, OXG5-AM, and OXG6-AM could maintain good system stability over time during storage, directly indicating that this system has excellent resistance to natural sedimentation. This is an important industrial performance indicator for acidic milk beverages and is highly consistent with the CSR results that reflect the aggregation trend of milk proteins.

The observed gradient in visual stability can be explained by differences in the casein–polysaccharide interaction mechanism, which is governed by the charge characteristics and particle size of the stabilizer [5]. The rigid double-helix structure and specific charge distribution of native XG hinder the formation of a stable complex with casein via flexible chain winding or charge complementarity under acidic conditions, leading to phase separation and casein precipitation. For OXG1, OXG2, and OXG3, the limited number of carboxyl groups may lead to relatively weak electrostatic interactions with casein. Furthermore, their larger particle size may limit close adhesion to the surface of casein protein, which may not be sufficient to effectively inhibit the collision and aggregation of protein particles. Over time, this leads to the formation of large protein aggregates that eventually sediment under gravity. In contrast, the high-DS OXG (OXG4–OXG6), with their increased carboxyl content and reduced particle size, can form a charged protective layer on the casein surface through electrostatic adsorption. Simultaneously, they are unfavorable to limit particle aggregation and particle size growth by means of spatial steric hindrance and electrostatic repulsion, potentially maintaining the particles in a stable colloidal size range [59]. This significantly reduces the tendency of gravity sedimentation, and thus there is no obvious whey separation or precipitation during the 15 d storage period. This observation further highlights the effectiveness of TEMPO oxidation as a strategy to enhance the stabilizing performance of XG in acidic protein systems.

### 3.6. Determination of Viscosity and Rheological Properties of AMDs

The viscosity of AMD samples as a function of shear rate is presented in Figure 5B. The apparent viscosity data of AMDs are shown in Table 3. The viscosities of AMD samples with XG and OXG1-6 as stabilizers were 124.000 ± 3.800 mPa·s, 107.800 ± 3.100 mPa·s, 94.200 ± 3.900 mPa·s, 83.400 ± 2.100 mPa·s, 20.100 ± 0.800 mPa·s, 14.900 ± 0.300 mPa·s, and 12.800 ± 0.600 mPa·s, respectively. The viscosity of CMC-AM was 13.000 ± 0.400 mPa·s. The results show that the AMD samples exhibit shear-thinning behavior within the shear rate range of 0.1 s^−1^ to 1000 s^−1^. The apparent viscosity decreased with increasing shear rate, eventually approaching a plateau at higher shear rates. The differences in viscosity among the samples were most pronounced at low shear rates. This behavior is typical of shear-thinning fluids and can be explained by the shear-induced disruption of weak physical interactions (e.g., hydrogen bonds, van der Waals forces) that maintain the three-dimensional network of protein aggregates. This disruption leads to aggregate disintegration, reduced internal flow resistance, and a subsequent decrease in viscosity until a steady state is reached [60,61]. XG-AM and samples with low-DS OXG (OXG1-AM, OXG2-AM, OXG3-AM) displayed substantially higher viscosities. This can be attributed to the formation of a gel-like network by XG in the AMD system, or to a higher degree of protein aggregation, both of which result in greater resistance to shear flow [62]. In contrast, AMD samples with higher-DS OXG (OXG4-AM, OXG5-AM, OXG6-AM) exhibited lower viscosities, with their flow curves progressively approaching that of CMC-AM. The viscosity change trends of AMD samples with different stabilizers are consistent with the viscosity change trends of OXG itself with different DS. AMD samples stabilized with higher-DS OXG exhibited lower apparent viscosity. This is likely the result of the combined effect of the apparent viscosity of the polysaccharide and the protein flocculation degree. In summary, the AMD samples with lower apparent viscosity have a relatively lower protein aggregation degree, a more gradual attenuation of shear-induced resistance in the rheological behavior, and better system stability. The viscosities of OXG5-AM and OXG6-AM were not significantly different from that of CMC-AM. This indicates that high-DS OXG can achieve effective stabilization without imparting excessive viscosity, a significant advantage for its potential application as a direct substitute for CMC in industrial formulations.

### 3.7. Particle Size Distribution of AMDs

The particle size distribution of the protein aggregates in AMDs was determined using laser diffraction, and the results are presented in Figure 5C. The particle size distribution of AMDs showed three distinct regions: a large-particle region (>10 μm), a medium-particle region (1–10 μm), and a small-particle region (<1 μm). A systematic shift in the particle size distribution was observed when XG was replaced with OXG, and this shift became more pronounced with increasing DS. XG-AM, OXG1-AM, OXG2-AM and OXG3-AM exhibited a broad particle size distribution with prominent peaks in the large-particle region (>10 μm), indicating extensive phase separation and the formation of large protein flocs under acidic conditions. With increasing DS of OXG (OXG4-AM to OXG6-AM), the proportion of large aggregates significantly decreased, and the distribution shifted towards smaller particle sizes. The intensity of the peak in the small-particle region (<1 μm) increased progressively. Notably, within the small-particle peak distribution range, the small-particle peak of AMDs with OXG6 added was higher than that with CMC added. The observed shift in particle size distribution might be partly attributed to the increased carboxyl content of OXG at higher DS, which strengthens electrostatic interactions with positively charged casein. Based on the variation patterns of particle size distribution, as well as the results of CSR and turbidity, it can be further clarified that the ability of polysaccharides to stabilize casein is related to the content of polysaccharide–casein complexes and the repulsive force between the complexes, and this process is highly correlated with the structural characteristics of the polysaccharide molecular weight. Previous studies have shown that the mechanism of stabilization can depend on molecular weight. For example, Tian et al. [63] reported that high-molecular-weight (1.42 × 10^6^ Da) soluble soybean polysaccharide (SSPS) stabilized casein primarily through steric hindrance, whereas a lower molecular weight fraction (8.8 × 10^5^ Da) adsorbed more readily onto casein surfaces via electrostatic interactions, forming more stabilizing complexes. Similarly, Ning Ma et al. [64] demonstrated that the lower molecular weight chitosan (150 kDa) forms strong electrostatic interactions with casein through sufficient protonated amino groups, and its binding constant is the highest, resulting in the strongest stability of the formed complex. However, the high-molecular-weight chitosan (300 kDa) causes casein precipitation due to hydrophobic self-aggregation. The large-molecule XG cleavage into small-molecule polysaccharides can improve the stability of acidic milk beverages, suggesting that the electrostatic interaction between oxidized XG and casein plays a dominant role, rather than the spatial steric hindrance effect produced by the long molecular chains. The shorter, more flexible OXG chains may adsorb more uniformly onto the surface of milk proteins, which could reduce the likelihood of bridging flocculation. This more uniform adsorption might enhance steric repulsion between protein-coated particles, thereby potentially inhibiting further aggregation and promoting a more homogeneous system.

### 3.8. Confocal Laser Scanning Microscope (CLSM) Analysis

**Figure 6:**
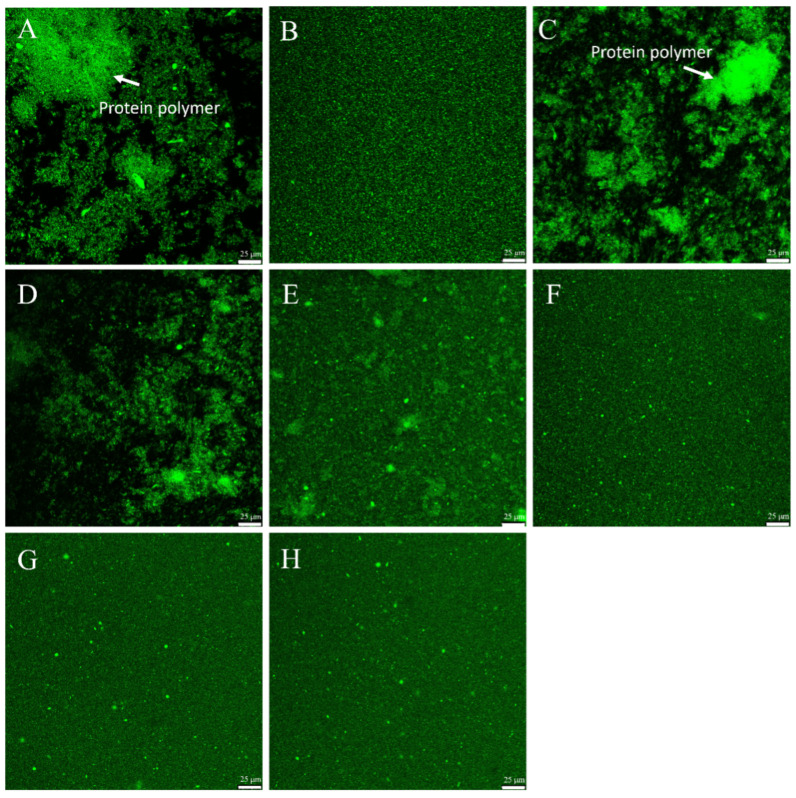
CLSM images illustrating the effect of different polysaccharides on the protein aggregation microstructure in AMDs. (**A**) XG-AM. (**B**) CMC-AM. (**C**–**H**) are respectively OXG1-AM, OXG2-AM, OXG3-AM, OXG4-AM, OXG5-AM, and OXG6-AM.

The microstructure of the AMD samples was examined using CLSM analysis to visualize the distribution and aggregation state of proteins. Proteins were stained with Fast Green FCF and appear green in the CLSM images as shown in Figure 6. As shown in Figure 6A,C, AMDs stabilized with XG or OXG1 exhibited large, irregular protein aggregates separated by extensive interconnected void regions, indicating severe phase separation. Under these acidic conditions, with the pH near the isoelectric point of casein, the net charge on the proteins is minimal. The limited negative charge from XG and low-DS OXG is insufficient to stabilize the proteins, leading to extensive aggregation via charge neutralization and bridging flocculation [65]. This is consistent with the particle size measurement results. When OXG2 and OXG3 were used, the size of the protein aggregates decreased, as shown in Figure 6D,E. These samples showed a mixture of medium-sized aggregates and some finer protein particles. This suggests that introduction of a moderate number of carboxyl groups may allow for weak electrostatic binding to the positively charged casein surface. However, the negative charge on the surface of the complex appears insufficient, and the resulting weak electrostatic repulsion is not enough to disperse the protein aggregates. In contrast, for CMC (Figure 6B) and the high-DS OXG samples (OXG4–OXG6, Figure 6F–H), the protein aggregates were much smaller and densely, uniformly dispersed throughout the solution, indicating a stable, well-distributed system. The high degree of oxidation introduces a sufficient number of carboxyl groups, which greatly enhances electrostatic interactions with the positively charged proteins. Furthermore, the oxidation-induced changes in conformation including the loosening of the double helix and chain shortening increase the flexibility of the OXG molecules. This may allow them to wrap more uniformly around the protein surface. This more uniform coverage, combined with the high negative charge, may contribute to electrostatic and steric stabilization, thereby helping to inhibit further aggregation and resulting in a stabilizing effect comparable to that of CMC [66].

### 3.9. Scanning Electron Microscopy (SEM) Analysis

**Figure 7:**
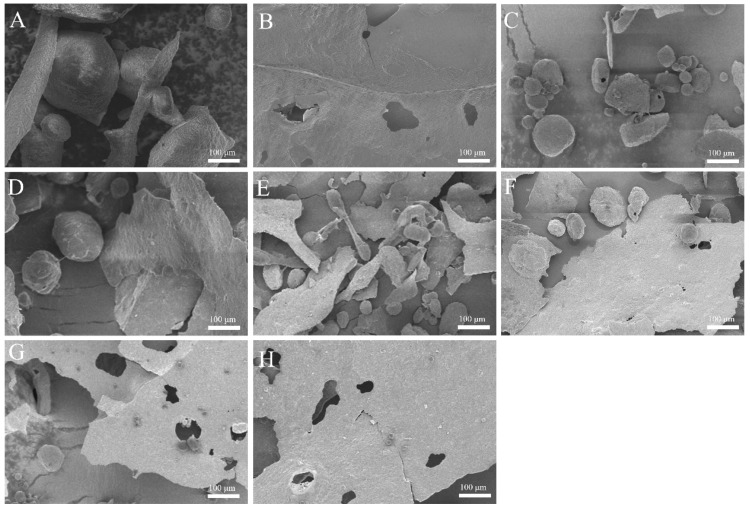
SEM images of the microstructure of freeze-dried AMDs supplemented with different polysaccharides. (**A**) XG-AM. (**B**) CMC-AM. (**C**–**H**) are respectively OXG1-AM, OXG2-AM, OXG3-AM, OXG4-AM, OXG5-AM, and OXG6-AM.

SEM was used to further examine the microstructure of the freeze-dried AMD samples, revealing the impact of different stabilizers on the protein–polysaccharide network. The micrographs are shown in Figure 7. The XG-AM sample is shown in Figure 7A, which exhibited a particle aggregate structure with non-uniform shape and size, relatively large particles, and a rough surface. This morphology is indicative of large-scale phase separation and protein flocculation, consistent with the instability of this sample. In contrast, the CMC-AM sample (Figure 7B) displayed a more continuous, smooth, and porous lamellar structure, characteristic of a well-stabilized system. With increasing oxidation degree, a systematic improvement in the microstructure was observed. The OXG1-AM sample (Figure 7C) still showed irregular aggregates, but with a noticeably smaller particle size compared to XG-AM. OXG2-AM, OXG3-AM, and OXG4-AM (Figure 7D–F) exhibited a transition towards a more sheet-like structure, albeit with poor integrity, and a further reduction in the number of irregular aggregates. The samples with the highest DS, OXG5-AM and OXG6-AM (Figure 7G,H), displayed a well-defined, continuous, and smooth lamellar structure. Moreover, the number of pores in the lamellar structure increased, and the proportion of irregular aggregates further decreased. Their morphology closely resembled that of the stable CMC-AM samples. The porous sheet-like structure may provide better support during the freeze-drying process, ensuring that the sample remains relatively intact. The SEM results provide microscopic evidence that TEMPO oxidation improves the compatibility of XG with the AMD system, leading to enhanced stability, a finding that aligns well with the macroscopic stability observations. Zhao et al. [67] investigated the effects of agaroligosaccharide (AO) and other polysaccharides in combination as stabilizers on the particle size and microscopic morphology of AMD particles. The results showed that when AO was used alone, the particle size of AMDs was the largest, and the stability of the system was poor. After freeze-drying treatment, its microscopic structure presented a loose and uneven network structure without a regular framework structure. However, when AO was combined with other anionic polysaccharides, it could effectively reduce the particle size of acidic milk beverages. This finding is consistent with our suggestion that the formation of a well-structured, porous network may be associated with enhanced stability in AMD systems.

## 4. Conclusions

This study aimed to address the poor compatibility of xanthan gum (XG) with milk proteins, which leads to phase separation in AMDs. A series of OXG samples with varying degrees of oxidation were successfully prepared using the TEMPO/NaClO/NaBr oxidation system. Their molecular structure and physicochemical properties were characterized using FT-IR, XRD, and GPC techniques. The stabilizing effect of OXG in AMDs and the underlying mechanism were investigated through a combination of techniques, including centrifugal sedimentation rate, turbidity, zeta potential, particle size analysis, CLSM, and SEM. The results showed that with increasing oxidation degree, the carboxyl substitution degree and uronic acid content of OXG increased significantly, whereas its molecular weight, apparent viscosity, and particle size decreased markedly. The negative zeta potential also became stronger. High-oxidation-degree OXG (OXG4–OXG6) was found to likely form a charged protective layer on the surface of milk proteins through a synergistic mechanism of electrostatic adsorption and steric hindrance. This may have effectively inhibited protein aggregation, as evidenced by a reduction in the centrifugal sedimentation rate of AMDs to as low as 2.22% and a zeta potential comparable to that of the CMC-stabilized control. Furthermore, it reduced system viscosity and promoted the formation of fine, dense protein–polysaccharide aggregates, thereby significantly improving the storage stability of AMDs. This study suggests that TEMPO-mediated oxidation can directionally regulate the structure and physicochemical properties of XG. The high-oxidation-degree OXG prepared in this study exhibited a stabilizing effect comparable to that of CMC, without the need for additional hydrocolloids. This finding provides a new theoretical basis and technical pathway for the modification of polysaccharides and their application in stabilizing acidic protein beverages. However, the proposed stabilization mechanism of “electrostatic adsorption–steric hindrance” is inferred from indirect physicochemical and microstructural observations. Direct quantitative verification, such as determination of protein–polysaccharide binding constants and adsorbed layer thickness using techniques like isothermal titration calorimetry, remains to be conducted. Future research will prioritize the use of such techniques to further validate the interaction mechanism of polysaccharide–protein and will focus on optimizing the industrial-scale preparation of OXG and exploring its application in plant-based acidic protein beverages, thereby providing more systematic theoretical support for practical applications.

## Figures and Tables

**Table 1:** AMD formulations with different polysaccharide stabilizers added.

Component/Units	Samples							
	CMC-AM	XG-AM	OXG1-AM	OXG2-AM	OXG3-AM	OXG4-AM	OXG5-AM	OXG6-AM
Skimmed milk powder/g	8.5	8.5	8.5	8.5	8.5	8.5	8.5	8.5
CMC/g	1							
XG/g		1						
OXG1/g			1					
OXG2/g				1				
OXG3/g					1			
OXG4/g						1		
OXG5/g							1	
OXG6/g								1
10% citric acid/mL	8	8	8	8	8	8	8	8
Potassium sorbate/g	0.6	0.6	0.6	0.6	0.6	0.6	0.6	0.6
Total amount/mL	200	200	200	200	200	200	200	200

Note: CMC stands for sodium carboxymethyl cellulose, XG for xanthan gum, and OXG1, OXG2, OXG3, OXG4, OXG5, and OXG6 respectively represent xanthan gum with different degrees of TEMPO oxidation. XG-AM indicates AMDs with xanthan gum added, and CMC-AM indicates AMDs with sodium carboxymethyl cellulose added. OXG1-AM, OXG2-AM, OXG3-AM, OXG4-AM, OXG5-AM, and OXG6-AM indicate AMDs with xanthan gum of different TEMPO oxidation degrees.

**Table 2:** Substitution degree, uronic acid content, molecular weight, viscosity, zeta potential and particle size of XG and OXG.

Samples	DS	Uronic Acid Content (μg/mL)	M_w_ (10^6^ g/mol)	Viscosity (mPa·s)	Zeta Potential (mV)	Particle Size (nm)
XG	0.174 ± 0.004 g	49.805 ± 0.065 g	9.610 ± 0.080 a	1609.000 ± 15.000 a	−66.600 ± 2.100 a	3016.000 ± 443.000 a
OXG1	0.185 ± 0.003 f	51.375 ± 0.025 f	7.660 ± 0.060 b	1549.000 ± 39.000 a	−72.300 ± 1.300 b	2870.000 ± 86.000 a
OXG2	0.192 ± 0.001 e	52.851 ± 0.021 e	5.830 ± 0.010 c	1379.000 ± 59.000 b	−72.800 ± 2.100 b	2669.000 ± 231.000 a
OXG3	0.211 ± 0.003 d	53.061 ± 0.043 d	2.980 ± 0.010 d	1026.000 ± 38.000 c	−74.000 ± 2.400 b	2249.000 ± 21.0003 b
OXG4	0.233 ± 0.001 c	53.425 ± 0.013 c	1.420 ± 0.010 e	744.000 ± 64.000 d	−78.400 ± 0.400 bc	1488.000 ± 96.000 c
OXG5	0.248 ± 0.003 b	54.174 ± 0.021 b	1.280 ± 0.010 f	498.000 ± 14.000 e	−73.800 ± 1.000 b	856.000 ± 58.000 d
OXG6	0.269 ± 0.001 a	54.747 ± 0.051 a	0.580 ± 0.010 g	436.000 ± 20.000 e	−72.800 ± 0.100 b	650.000 ± 32.000 d

Note: Different lowercase letters in the same column indicate significant differences (*p* < 0.05). “XG” stands for natural xanthan gum, “OXG1, OXG2, OXG3, OXG4, OXG5, OXG6” represent TEMPO-oxidized xanthan gum, and “DS” represents the degree of substitution.

**Table 3:** Centrifugal precipitation rate, turbidity, viscosity, and zeta potential measurements of AMDs.

Measurement Index	AMD Samples							
	XG-AM	CMC-AM	OXG1-AM	OXG2-AM	OXG3-AM	OXG4-AM	OXG5-AM	OXG6-AM
CSR(%)	8.930 ± 0.070 a *	2.050 ± 0.260 g	6.850 ± 0.060 b *	5.990 ± 0.140 c *	5.480 ± 0.080 d *	4.660 ± 0.040 e *	2.540 ± 0.020 f *	2.220 ± 0.010 g
Turbidity	0.040 ± 0.020 f *	1.110 ± 0.060 bc	0.700 ± 0.040 e *	0.910 ± 0.030 d *	0.950 ± 0.03 d *	1.060 ± 0.010 c	1.110 ± 0.020 b	1.330 ± 0.020 a *
Viscosity (mPa·s)	124.000 ± 3.800 a *	13.000 ± 0.400 f	107.800 ± 3.100 b *	94.200 ± 3.900 c *	83.400 ± 2.100 d *	20.100 ± 0.800 e *	14.900 ± 0.300 f	12.800 ± 0.600 f
Zeta potential (mV)	−32.600 ± 0.300 g *	−38.000 ± 0.500 h	−33.500 ± 0.100 f *	−34.300 ± 0.200 e *	−34.900 ± 0.200 d *	−35.900 ± 0.100 c *	−36.700 ± 0.100 b *	−37.900 ± 0.300 a

Note: Different lowercase letters in the same line indicate significant differences (*p* < 0.05); * indicates significant differences compared with CMC-AM (*p* < 0.05); without * indicates no significant difference compared with CMC-AM. Among them, XG-AM represents AMDs with xanthan gum added, CMC-AM represents AMDs with sodium carboxymethyl cellulose added, and OXG1-AM, OXG2-AM, OXG3-AM, OXG4-AM, OXG5-AM, and OXG6-AM represent AMDs with xanthan gum of different oxidation degrees of TEMPO added.

## Data Availability

The original contributions presented in this study are included in the article. Further inquiries can be directed to the corresponding authors.

## Abbreviations

The following abbreviations are used in this manuscript:

AMD	Acidified milk drink
CLSM	Confocal laser scanning microscope
CSR	Centrifugal sedimentation rate
DS	Degree of substitution
FT-IR	Fourier transform infrared spectroscopy
G′	Storage modulus
G″	Loss modulus
GPC	Gel permeation chromatography
OXG	Oxidized xanthan gum
PDI	Polydispersity index
SEM	Scanning electron microscope
TEMPO	2,2,6,6-tetramethylpiperidin-1-oxyl
XRD	X-ray diffraction
XG	Xanthan gum
